# Heterogeneous Nucleation of Trichloroethylene Ozonation Products in the Formation of New Fine Particles

**DOI:** 10.1038/srep42600

**Published:** 2017-02-15

**Authors:** Ning Wang, Xiaomin Sun, Jianmin Chen, Xiang Li

**Affiliations:** 1 Environment Research Institute, Shandong University, Jinan 250100, P. R. China; 2 Department of Environmental Science and Engineering, Fudan University, China

## Abstract

Free radicals in atmosphere have played an important role in the atmospheric chemistry. The chloro-Criegee free radicals are produced easily in the decomposition of primary ozonide (POZ) of the trichloroethylene, and can react with O_2_, NO, NO_2,_ SO_2_ and H_2_O subsequently. Then the inorganic salts, polar organic nitrogen and organic sulfur compounds, oxygen-containing heterocyclic intermediates and polyhydroxy compounds can be obtained. The heterogeneous nucleation of oxidation intermediates in the formation of fine particles is investigated using molecular dynamics simulation. The detailed nucleation processes are reported. According to molecular dynamics simulation, the nucleation with a diameter of 2 nm is formed in the Organic Compounds-(NH_4_)_2_SO_4_-H_2_O system. The spontaneous nucleation is an important process in the formation of fine particles in atmosphere. The model study gives a good example from volatile organic compounds to new fine particles.

Atmospheric organic aerosols have enormous and remarkable impact on the global climate. They influence the formation and lifetime of clouds, and affect the concentration of gas phase species as these species can react on aerosols’ surfaces^1^. Secondary organic aerosols (SOA) account for approximately 10% of the total organic aerosol loadings and up to 90% of organic aerosols in urban environments^2^.

The ozonolysis of alkenes is an important reaction in atmospheric chemistry, and ozonolysis intermediates-Criegee Intermediates (CIs) can be generated in this progress^3^,^4^. CIs will further generate the secondary ozonide (SOZ), which can make significant contribution to SOA mass, a significant source of SOA in the troposphere^5^,^6^,^7^,^8^,^9^,^10^.

Previous studies suggest that aerosols, fog, and cloud water may play a key role in the atmospheric chemistry^11^,^12^,^13^. In-cloud SOA formation is likely to enhance organic PM concentrations in the free troposphere and organic aerosol concentrations in locations, and leads to serious pollution in the atmospheric environment. There are several significant atmospheric events, such as London Fog mainly emanated by SO_2_ from coal burning^14^, Los Angeles Smog caused by NOx and VOCs from traffic emissions^15^,^16^,^17^, Beijing Haze co-initiated by SO_2_, NOx and VOCs^18^,^19^,^20^. One of the most important properties is the high level of particulate matter which profoundly impacts human health, visibility, the ecosystem, weather and climate^21^,^22^.

So far, the mechanisms of PM formation remain uncertain in detail, especially for microscopic mechanism of the processes related to PM origin and growth^2^,^23^,^24^,^25^,^26^. A few theoretical researches focuses on PM formation mechanism which involves many chemical constituents of organic and elemental carbon, sulfate, nitrate, ammonium and trace metals carbon.

It is well known that oxidants play a crucial role in atmospheric chemistry processes. Free radicals produced by the olefin’s ozonation become a major kind of atmospheric oxidants which is key to the formation of fine particles, and has drawn great attention^27^,^28^,^29^,^30^.

Trichloroethylene (TCE), as a kind of chloroethylenes, is one of the most important volatile organic compounds (VOCs)^31^,^32^. TCE is a contaminant as it is highly toxic, carcinogenic, when it is released into the atmosphere and subsurface of groundwater^33^,^34^,^35^.

In the ozonolysis process of TCE, a primary ozonide (POZ) is produced. It is a cyclic compound and will decompose into two kinds of CI (i.e., IM1 and IM2), phosgene and formyl chloride, eventually produce two kinds of SOZ (i.e., SOZ1 and SOZ2). SOZ can make significant contribution to SOA mass.

In this paper, the oxidation intermediates of chlorinated Criegee radicals produced in trichloroethylene ozonation on the heterogeneous nucleation of the fine particles is under investigation using molecular simulation. The molecular dynamics simulation will be performed to study nucleation in the OCs-(NH_4_)_2_SO_4_-H_2_O system, which is an important process in the formation of fine particles in atmosphere.

## Results

### The new particle formation

Many polar intermediates will be formed in the process of trichloroethylene ozonation, such as POZ, SOZ*i (i* = 1–2), IM*i (i* = 1–20), and P*i (i* = 1–14) (See Supplementary Fig. S1-S3). These species will participate in the nucleation of new particles or the formation of secondary organic aerosols through their own aggregation or absorbing fine particles in the atmosphere. The systems consisting of above typical Organic Compounds(OC), (NH_4_)_2_SO_4_, and H_2_O are chosen as an example to study equilibrium distributions of individual particles using molecular dynamics simulation and to further probe the formation of new fine particles. The typical OCs are POZ, SOZ1, IM6, IM11, P2, P3, P6, P7 and P10.

### Distribution of equilibrium configurations

**Figure 1:**
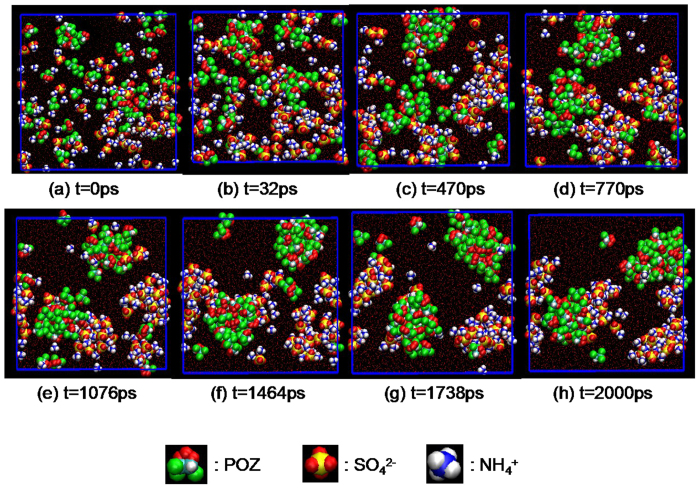
The snapshot of distribution of the equilibrium configuration in the POZ-(NH_4_)_2_SO_4_-H_2_O system at different time.

The snapshots of POZ-(NH_4_)_2_SO_4_-H_2_O system at different time are discussed as an example in Fig. 1. At t = 0 ps, there is no obvious nucleation and the particles are dispersed uniformly. With simulation time increasing, nucleation phenomenon is more and more evident. When the system reaches equilibrium, both POZ and (NH_4_)_2_SO_4_ have a nucleus solely and larger clusters through their absorption of each other. Other systems reveal the similar phenomenon, and snapshots for the distribution of equilibrium configuration (see Supplementary Fig. S4–S12). Each system can form clusters of different sizes, *i.e.*, different nucleus, including inter- and intra- nucleation of the (NH_4_)_2_SO_4_ and OCs. The diameter of the nucleation clusters is about 2 nm.

### Radial distribution function

For further data analysis, the radial distribution function of those particles can be obtained from the thermodynamic equilibrium system. The Radial distribution function *g (r)* is a reflection on the physical characteristics of the fluid microstructure, which indicates a probability of another molecule density around a molecule at a distance *r* where the ratio is randomly distributed with respect to the probability density.


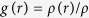


where *ρ(r)* is the local density from the center of molecule *r* to the volume element *dr*, and *ρ* is the average density.

**Figure 2:**
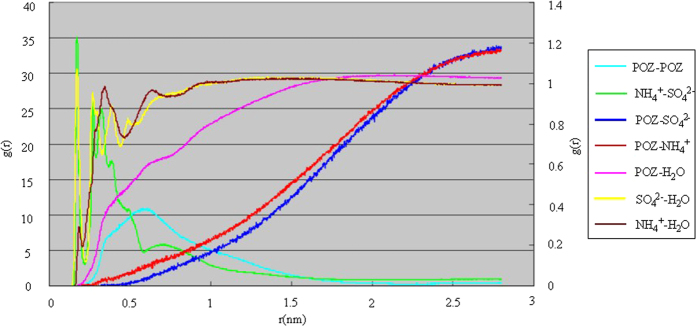
The radial distribution functions for the main particles in the POZ-(NH_4_)_2_SO_4_-H_2_O system.

The radial distribution functions for main particles in the POZ-(NH_4_)_2_SO_4_-H_2_O system are suggested in Fig. 2. It is obvious that the radial distribution function curve of NH_4_^+^ and SO_4_^2−^ have two distribution peaks around 0.21 nm and 0.35 nm, mainly due to electrostatic interactions between NH_4_^+^ and SO_4_^2−^. In the RDF curve of NH_4_^+^ and H_2_O, there are two peaks about 0.35 nm and 0.61 nm too. The first peak is mainly resulted from the static electricity of NH_4_^+^ and H_2_O and the hydration structure (*i.e.*, the first hydration layer) could be generated. And the second peak comes from hydrogen bonds between the first hydration structure and other water to form the new hydration structure (*i.e.*, the second hydration layer). The RDF curve of SO_4_^2−^ and H_2_O is similar to that of NH_4_^+^ and H_2_O. The main difference lies in that peaks occur at smaller distance regions, which may be due to stronger Coulomb interaction between SO_4_^2−^ and H_2_O. Then the stronger Coulomb interaction can help the inner hydration layer water take effect with other water to produce the second and third hydration layer through hydrogen bonds. There are three distinct peaks in the RDF curve of SO_4_^2−^ and H_2_O. There is a clear and broad peak at 0.6 nm in the RDF curve of POZ and POZ, indicating the presence of nucleation between organic molecules. It can be found that the RDF curves of POZ and SO_4_^2−^, NH_4_^+^, H_2_O have a monotonously rising trend, which indicates that there is no obvious aggregation between POZ and SO_4_^2−^, NH_4_^+^, H_2_O. In addition, the RDF curves of POZ and H_2_O are steeper than those of POZ and SO_4_^2−^, NH_4_^+^. So the water molecules begin to have a significant position in distribution at a distance of 0.5–1.5 nm around POZ, while SO_4_^2−^, NH_4_^+^ at a distance of 2.25 nm. What accounts for this phenomenon is the appearance of hydration structures between SO_4_^2−^, NH_4_^+^ and H_2_O.

The radial distribution function of other systems described above (see Supplementary Fig. S13-S20), respectively. The radial distribution functions of NH_4_^+^-SO_4_^2−^, NH_4_^+^-H_2_O and SO_4_^2−^-H_2_O between (NH_4_)_2_SO_4_ and H_2_O show the same trend in different systems, but the main difference exists in the radial distribution functions of OCs-OCs, OCs-SO_4_^2−^ and OCs-H_2_O which have different peaks.

### The Binding mode and intermolecular force

**Figure 3:**
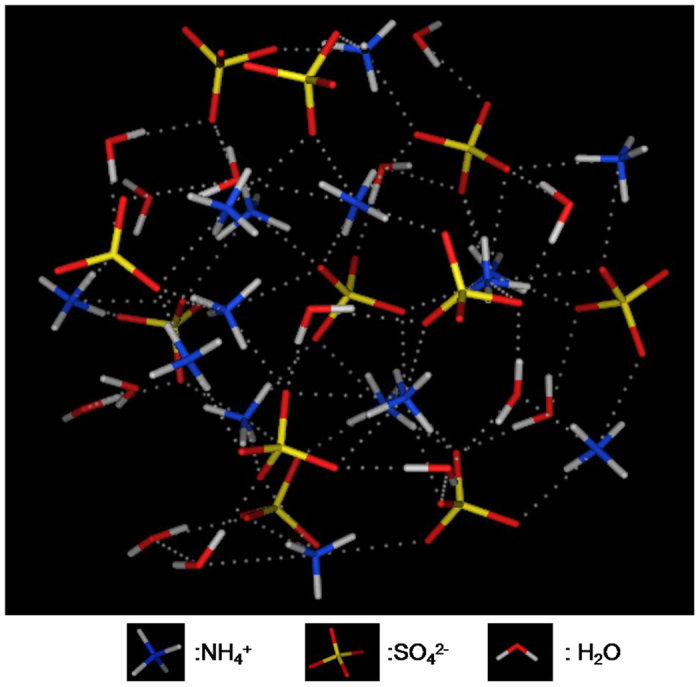
The bonding mode of SO_4_^2−^ with other particles around 0.5 nm in the POZ-(NH_4_)_2_SO_4_-H_2_O system.

**Figure 4:**
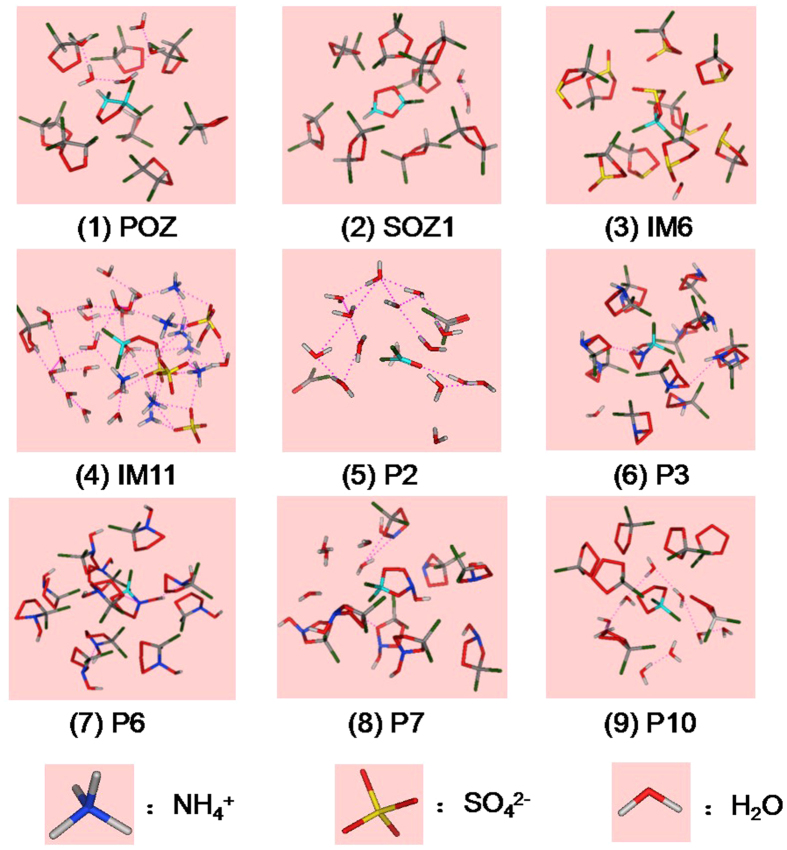
The binding mode of VOCs with other particles around 0.5 nm in the VOCs–(NH_4_)_2_SO_4_-H_2_O system.

The binding mode of SO_4_^2−^ with other particles in distance of 0.45 nm in the POZ-(NH_4_)_2_SO_4_-H_2_O system is drawn in Fig. 3. With SO_4_^2−^ as the center, a three-dimensional cage-like structure is formed with ammonium ion and water. The binding modes of different OCs are shown in Fig. 4. It is easy to see the distribution of different molecules around the center of OCs molecules. The water molecules are embedded in clusters of cyclic intermediates, for example, POZ, SOZ1, P3, P7, IM6, P6, and P10. As water soluble strong hydroxyl compounds, the IM11 can form the cluster with more water molecules, ammonium ion and sulfate ion. While P2 is mainly surrounded by water molecules. The forces between pairs of NH_4_^+^-SO_4_^2−^, NH_4_^+^-H_2_O and SO_4_^2−^-H_2_O are the charge electrostatic interaction, ion-induced and intermolecular hydrogen bonds. While the forces of OCs-OCs, OCs-SO_4_^2−^, OCs-H_2_O are referred to as the dispersion forces between polar molecules, ion-induced and intermolecular hydrogen bonds. The binding energies between different particle-pairs in the OCs-(NH_4_)_2_SO_4_-H_2_O system are listed in Table 1.

The main driving force for the formation of clusters, *i.e.*, nucleation, should be the overall performance of charge electrostatic interaction, ion induced, dispersion forces, and intermolecular hydrogen bonds, and other factors. The peak value of radial distribution function reflects the mutual attraction between the particles with nucleating ability. The higher the peak is, the easier the nucleation is. And the peak width of radial distribution function is able to reflect the nucleus particle size. The wider the peak is, the bigger the size of the formation particle is.

## Discussion

The Criegee radical generated in the ozonation of TCE can react with NO, NO_2_, SO_2_, H_2_O, O_2_ and other species in the atmosphere. During the oxidation process, the NO can be oxidized to NO_2_ and then to NO_3_, SO_2_ can also be oxidized to SO_3_, and then inorganic acid can be produced. In the presence of NH_3_, inorganic salts (NH_4_)_2_SO_4_ and NH_4_NO_3_ can be formed.

Apart from inorganic salts, the polar organic nitrogen and organic sulfur compounds, oxygen-containing heterocyclic intermediates and polyhydroxy compounds can be obtained too.

**Figure 5:**
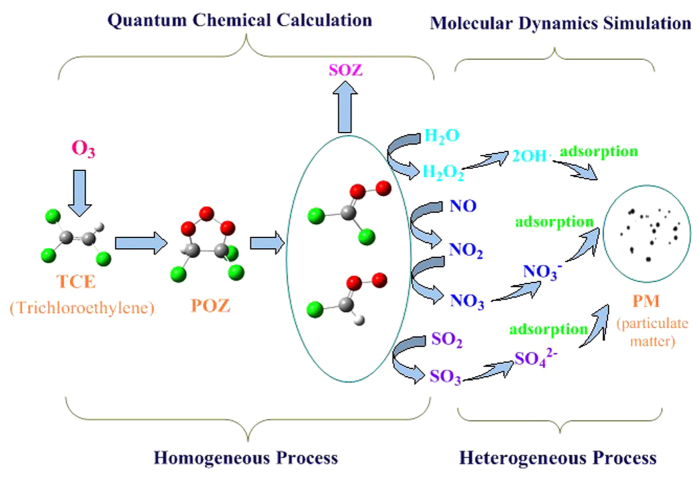
The schematic diagram from volatile organic compounds to the formation of new particle in the ozonation of trichloroethylene.

The polar organic intermediates, such as POZ, SOZ, organic nitrogen and organic sulfur compounds, oxygen-containing heterocyclic intermediates, and polyhydroxy compounds have their own tendency to aggregate to generate new fine particles and further produce the second organic aerosols in inorganic salt surface. The schematic diagram from volatile organic compounds to the formation of new particle in the ozonation of trichloroethylene is shown in Fig. 5.

## Computational methods

### Molecular Dynamic Simulation

The GROMACS 4.5.5 package with Amber99SB force field was used for all molecular dynamics simulations^36^. The GROMACS 4.5.5 package with Amber99SB force field was used for all molecular dynamics simulations^36^. The topology parameters of OCs were derived using RESP (Restrained Electro Static Potential) method with AM1-BCC model, which can create high quality atomic charges for organic molecules in polar solvent^37^. TIP3P model was used for water molecules. One OC molecule was placed in a box of 7 nm × 7 nm × 7 nm. Then 49 OCs, 50 SO_4_^2−^, 100 NH_4_^+^ and 5560 water molecules were inserted randomly into the box. Finally the system includes 50 OCs, 50 (NH_4_)_2_SO_4_ and 5560 water molecules. Energy minimization and 100 ps NVT MD simulation were carried out to remove the steric clash. The subsequent 200 ps NPT MD simulation was done to adjust the box size and achieve reasonable density. Production MD simulation was performed for 2 ns at 300 K and the time step is 1 fs. PME (Particle Mesh Ewald) method was used to consider long-range electrostatic interactions. The last 500 ps trajectory was used in the following analyses.

## Additional Information

**How to cite this article:** Wang, N. *et al*. Heterogeneous Nucleation of Trichloroethylene Ozonation Products in the Formation of New Fine Particles. *Sci. Rep.* **7**, 42600; doi: 10.1038/srep42600 (2017).

**Publisher's note:** Springer Nature remains neutral with regard to jurisdictional claims in published maps and institutional affiliations.

## Supplementary Material

Supplementary Information

## Figures and Tables

**Table 1:** The binding energy (kCal/mol) between different pair-particle in the OCs-(NH_4_)_2_SO_4_-H_2_O system.

		(N)-(N)	(N)-SO_4_^2−^	(N)-NH_4_^+^	(N)-SO_4_^2−^	SO_4_^2−^-NH_4_^+^	SO_4_^2−^-H_2_O	NH_4_^+^-H_2_O
N = POZ	Coul	−1.95	0.12	−0.821	−322.25	−6638.72	−4780.76	−1430.39
	LJ	−257.54	−0.74	−1.021	−33.27	504.51	308.55	259.05
	Total	−259.49	−0.61	−1.85	−355.52	−6134.21	−4472.21	−1171.34
N = SOZ1	Coul	−12.09	0.02	−0.32	−287.54	−7429.56	−4251.53	−1134.95
	LJ	−306.11	−0.54	−0.53	−30.44	564.58	269.04	216.81
	Total	−318.20	−0.52	−0.85	−317.97	−6864.98	−3982.50	−918.15
N = IM6	Coul	6.98	−0.76	−4.49	−366.94	−6764.85	−4694.14	−1401.29
	LJ	−223.38	−3.01	−2.21	−34.31	517.36	302.64	250.92
	Total	−216.41	−3.77	−6.70	−401.25	−6247.50	−4391.50	−1150.36
N = IM11	Coul	−229.33	−561.45	59.48	−268.54	−7529.78	−3598.82	−1235.42
	LJ	−26.02	30.07	−17.69	−29.55	581.41	212.04	218.16
	Total	−255.35	−531.38	41.80	−298.09	−6948.36	−3386.78	−1017.25
N = P2	Coul	−0.32	1.52	−7.51	−228.49	−6976.61	−4568.74	−1329.28
	LJ	−13.82	−2.91	−2.11	−326.75	531.24	296.48	245.05
	Total	−14.14	−1.39	−9.62	−555.24	−6445.37	−4272.26	−1084.23
N = P3	Coul	−6.037	−6.92	−1.67	−368.16	−7394.28	−4200.27	−1207.29
	LJ	−139.74	−3.58	−2.88	−31.38	558.69	272.55	222.94
	Total	−145.78	−10.49	−4.54	−399.54	−6835.59	−3927.71	−984.35
N = P6	Coul	−41.06	−155.41	7.22	−541.53	−6701.71	−4603.05	−1416.98
	LJ	−162.66	5.54	−8.03	−320.49	515.50	293.80	257.93
	Total	−203.72	−149.86	−0.81	−862.02	−6186.22	−4309.25	−1159.06
N = P7	Coul	40.056	−180.96	14.84	−328.14	−6842.47	−4506.65	−1427.18
	LJ	−189.13	8.74	−6.21	−33.71	520.11	286.34	253.76
	Total	−149.07	−172.23	8.63	−361.85	−6322.37	−4220.31	−1173.42
N = P10	Coul	1.89	0.19	−0.65	−23.31	−6416.25	−4995.53	−1501.96
	LJ	−254.31	−1.13	−1.09	−252.82	497.22	319.19	268.40
	Total	−252.42	−0.94	−1.74	−276.14	−5919.04	−4676.34	−1233.56
