# Supplementary material for: Heterogeneous Nucleation of Trichloroethylene Ozonation Products in the Formation of New Fine Particles
Source: Sci Rep. 2017 Feb 15;7:42600. doi: 10.1038/srep42600 (PMC5309831; doi:10.1038/srep42600)
Supplement: Supplementary Information [file srep42600-s1.pdf]

Supplementary Information for

Heterogeneous Nucleation of Trichloroethylene Ozonation Products in the Formation of New Fine Particles

Wang Ning[1], Xiaomin Sun[1*], Jianmin Chen[2], Xiang Li[2*]

[1]Environment Research Institute, Shandong University, Jinan 250100, P. R. China
[2] Department of Environmental Science and Engineering, Fudan University, China

Supplementary Figure S1 Schematic for the pathways of O_3 oxidation of TCE

Supplementary Figure S2 Schematic for the pathways of the reactions of IM1

Supplementary Figure S3 Schematic for the reaction pathways of IM2

Supplementary Figure S4 The snapshot of distribution of the equilibrium configuration in the POZ-$(NH_4)_2SO_4$-H_2O system.

Supplementary Figure S5 The snapshot of distribution of the equilibrium configuration in the SOZ1-$(NH_4)_2SO_4$-H_2O system.

Supplementary Figure S6 The snapshot of distribution of the equilibrium configuration in the IM6-$(NH_4)_2SO_4$-H_2O system.

Supplementary Figure S7 The snapshot of distribution of the equilibrium configuration in the IM11-$(NH_4)_2SO_4$-H_2O system.

Supplementary Figure S8 The snapshot of distribution of the equilibrium configuration in the P2-$(NH_4)_2SO_4$-H_2O system.

Supplementary Figure S9 The snapshot of distribution of the equilibrium configuration in the P3-$(NH_4)_2SO_4$-H_2O system.

Supplementary Figure S10 The snapshot of distribution of the equilibrium configuration in the P6-$(NH_4)_2SO_4$-H_2O system.

Supplementary Figure S11 The snapshot of distribution of the equilibrium configuration in the P7-$(NH_4)_2SO_4$-H_2O system.

Supplementary Figure S12 The snapshot of distribution of the equilibrium configuration in the P10-$(NH_4)_2SO_4$-H_2O system.

Supplementary Figure S13 The radial distribution functions for the main particles in the SOZ1-$(NH_4)_2SO_4$-H_2O system. (The left axis represents the g(r) of groups

SOZ1-SOZ1 and NH_4^+-SO_4^{2-} , the right axis represents the g(r) of the other groups)

Supplementary Figure S14 The radial distribution functions for the main particles in the IM6-$(NH_4)_2SO_4$-H_2O system. (The left axis represents the g(r) of groups IM6-IM6 and NH_4^+-SO_4^{2-} , the right axis represents the g(r) of the other groups)

Supplementary Figure S15 The radial distribution functions for the main particles in the IM11-$(NH_4)_2SO_4$-H_2O system. (The left axis represents the g(r) of groups IM11-IM11 and NH_4^+-SO_4^{2-} , the right axis represents the g(r) of the other groups)

Supplementary Figure S16 The radial distribution functions for the main particles in the P2-$(NH_4)_2SO_4$-H_2O system. (The left axis represents the g(r) of groups P2-P2 and NH_4^+-SO_4^{2-} , the right axis represents the g(r) of the other groups)

Supplementary Figure S17 The radial distribution functions for the main particles in the P3-$(NH_4)_2SO_4$-H_2O system. (The left axis represents the g(r) of groups P3-P3 and NH_4^+-SO_4^{2-} , the right axis represents the g(r) of the other groups)

Supplementary Figure S18 The radial distribution functions for the main particles in the P6-$(NH_4)_2SO_4$-H_2O system. (The left axis represents the g(r) of groups P6-P6 and NH_4^+-SO_4^{2-} , the right axis represents the g(r) of the other groups)

Supplementary Figure S19 The radial distribution functions for the main particles in the P7-$(NH_4)_2SO_4$-H_2O system. (The left axis represents the g(r) of groups P7-P7 and NH_4^+-SO_4^{2-} , the right axis represents the g(r) of the other groups)

Supplementary Figure S20 The radial distribution functions for the main particles in the P10-$(NH_4)_2SO_4$-H_2O system. (The left axis represents the g(r) of groups P10-P10 and NH_4^+-SO_4^{2-} , the right axis represents the g(r) of the other groups)

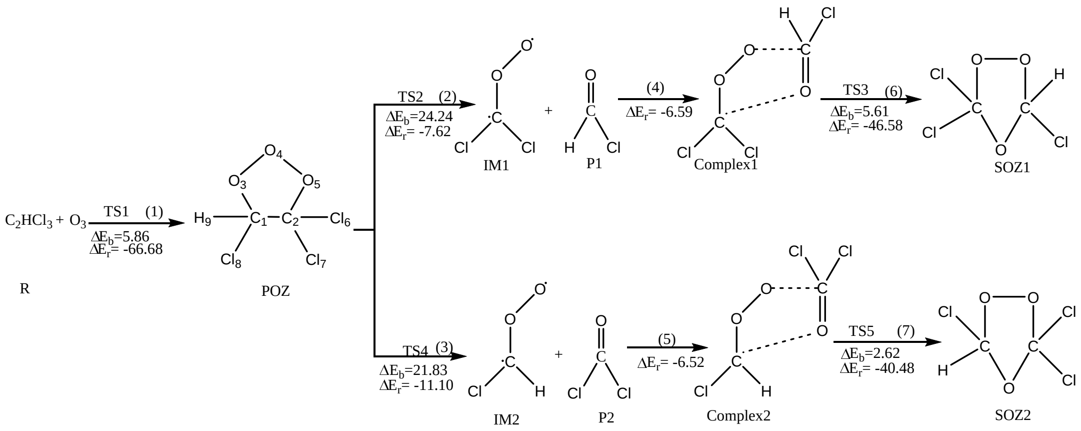

Supplementary Figure S1 Schematic for the pathways of O_3 oxidation of TCE

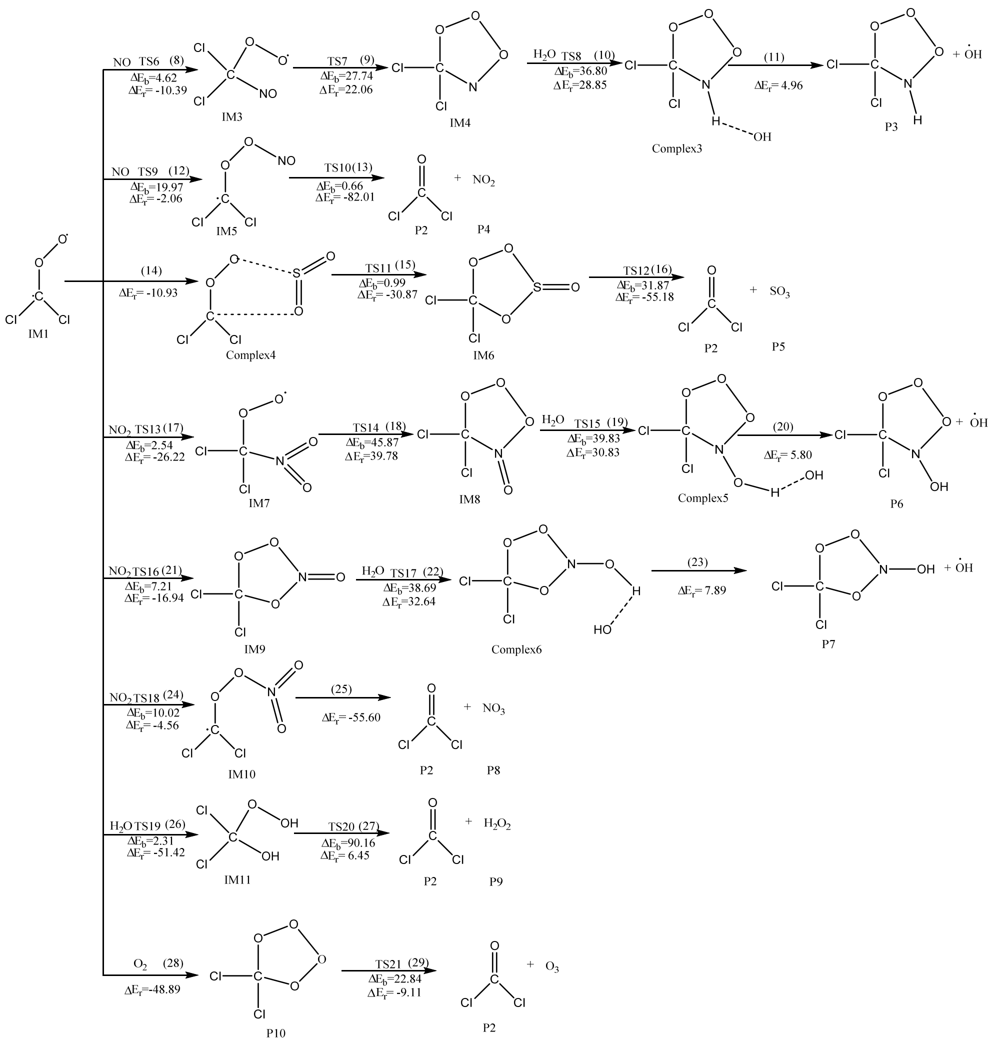

Supplementary Figure S2 Schematic for the pathways of the reactions of IM1

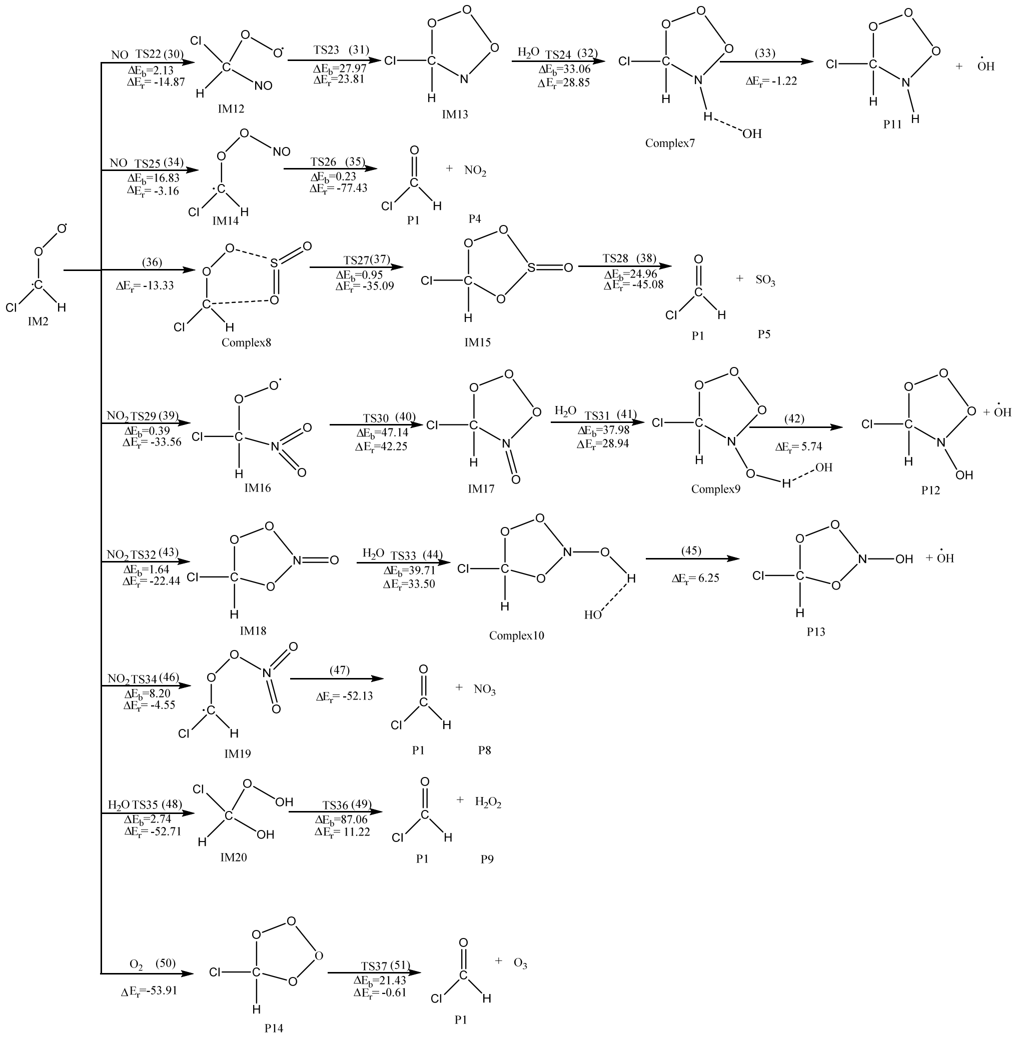

Supplementary Figure 3. Schematic for the reaction pathways of IM2

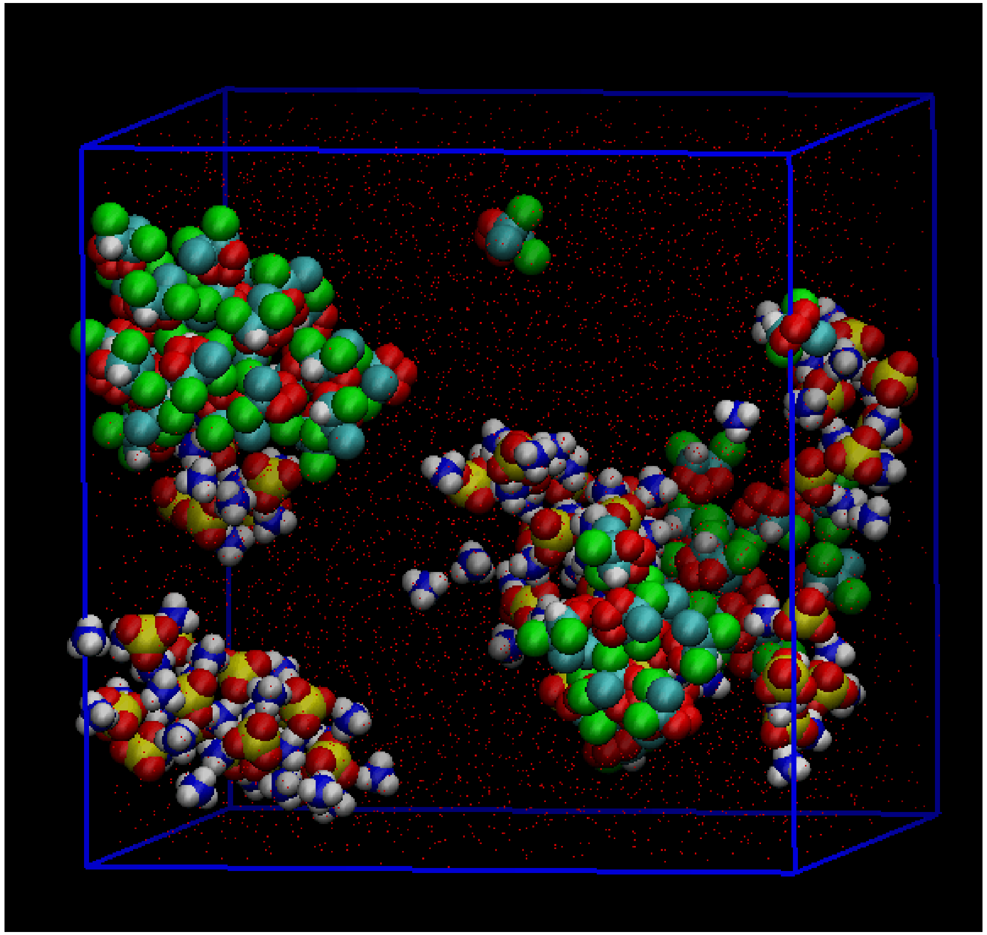

Supplementary Figure S4 The snapshot of distribution of the equilibrium configuration in the POZ-$(NH_4)_2SO_4$-H_2O system.

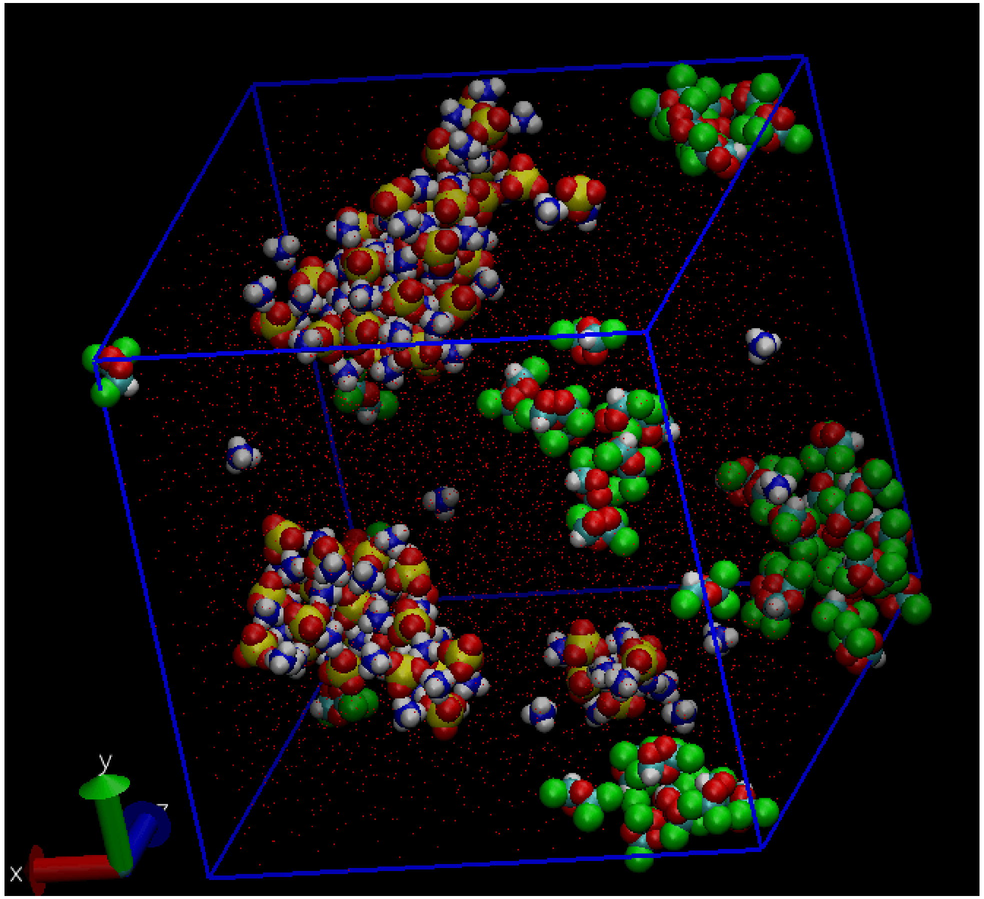

Supplementary Figure S5 The snapshot of distribution of the equilibrium configuration in the SOZ1-$(NH_4)_2SO_4$-H_2O system.

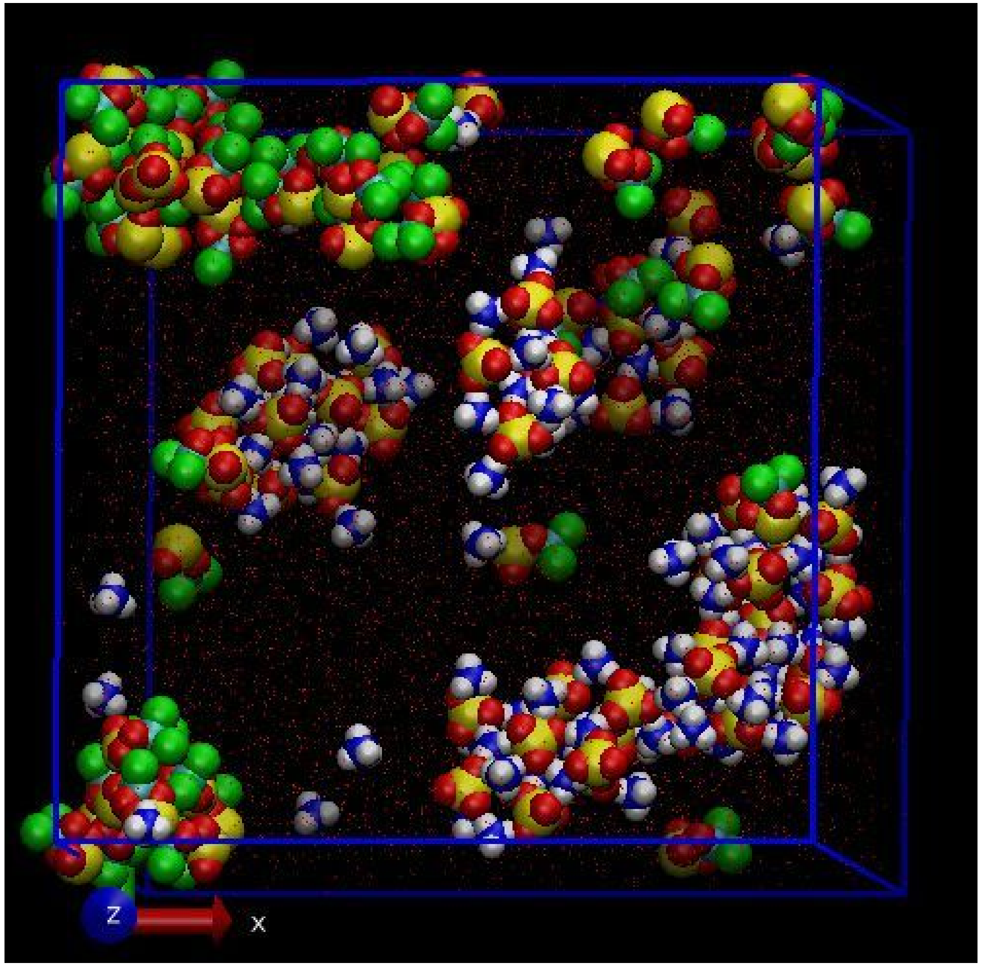

Supplementary Figure S6 The snapshot of distribution of the equilibrium configuration in the IM6-$(NH_4)_2SO_4$-H_2O system.

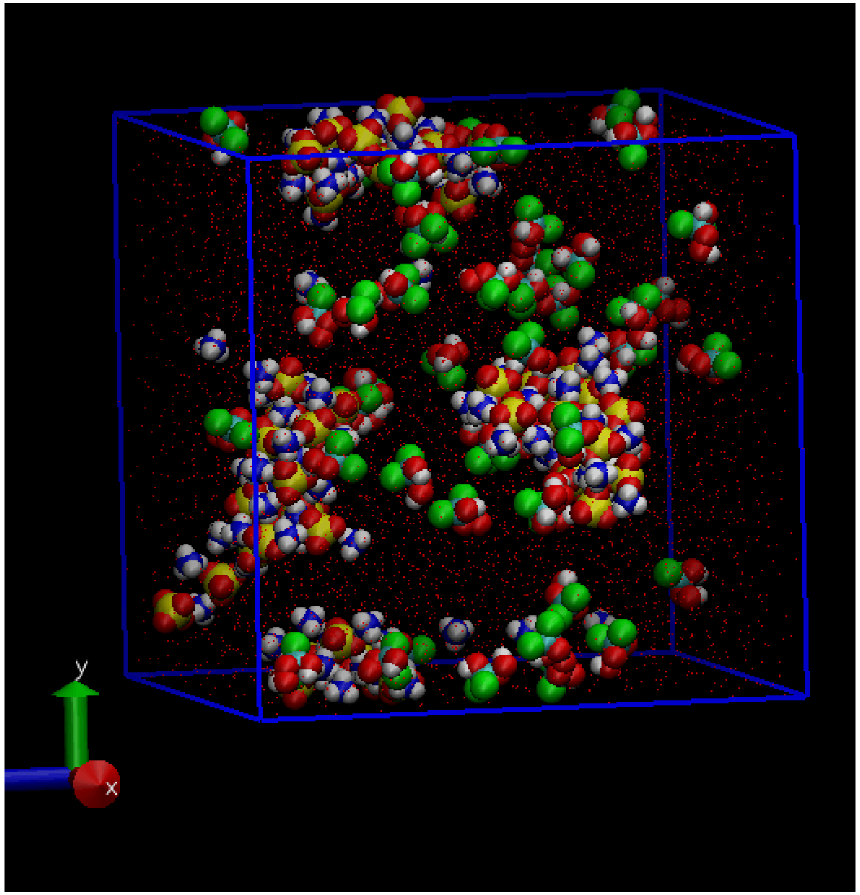

Supplementary Figure S7 The snapshot of distribution of the equilibrium configuration in the IM11-$(NH_4)_2SO_4$-H_2O system.

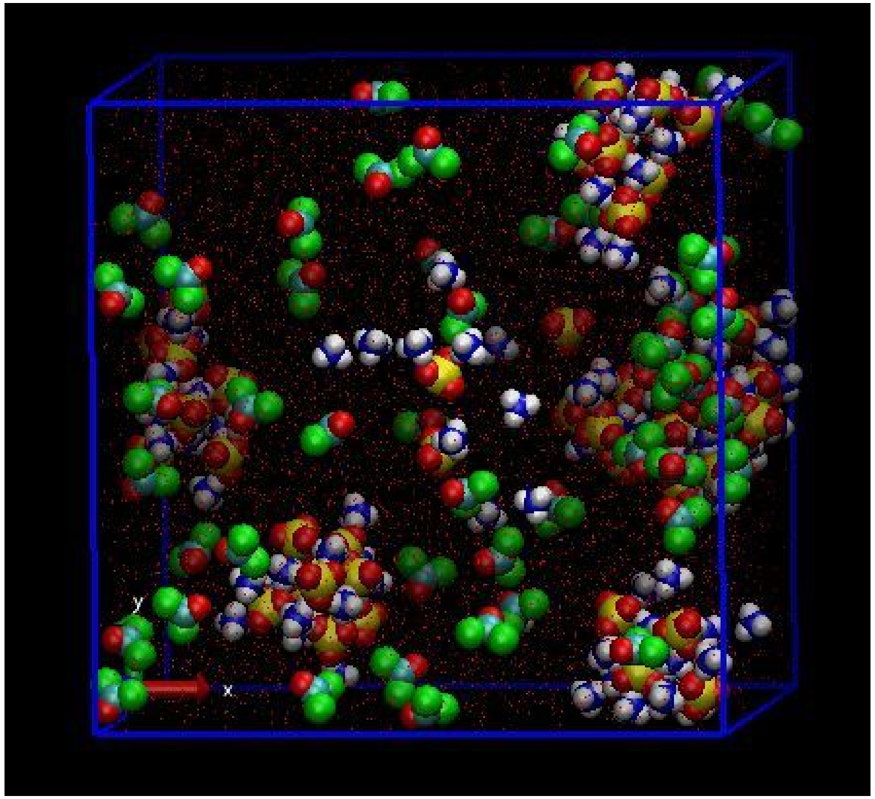

Supplementary Figure S8 The snapshot of distribution of the equilibrium configuration in the P2-$(NH_4)_2SO_4$-H_2O system.

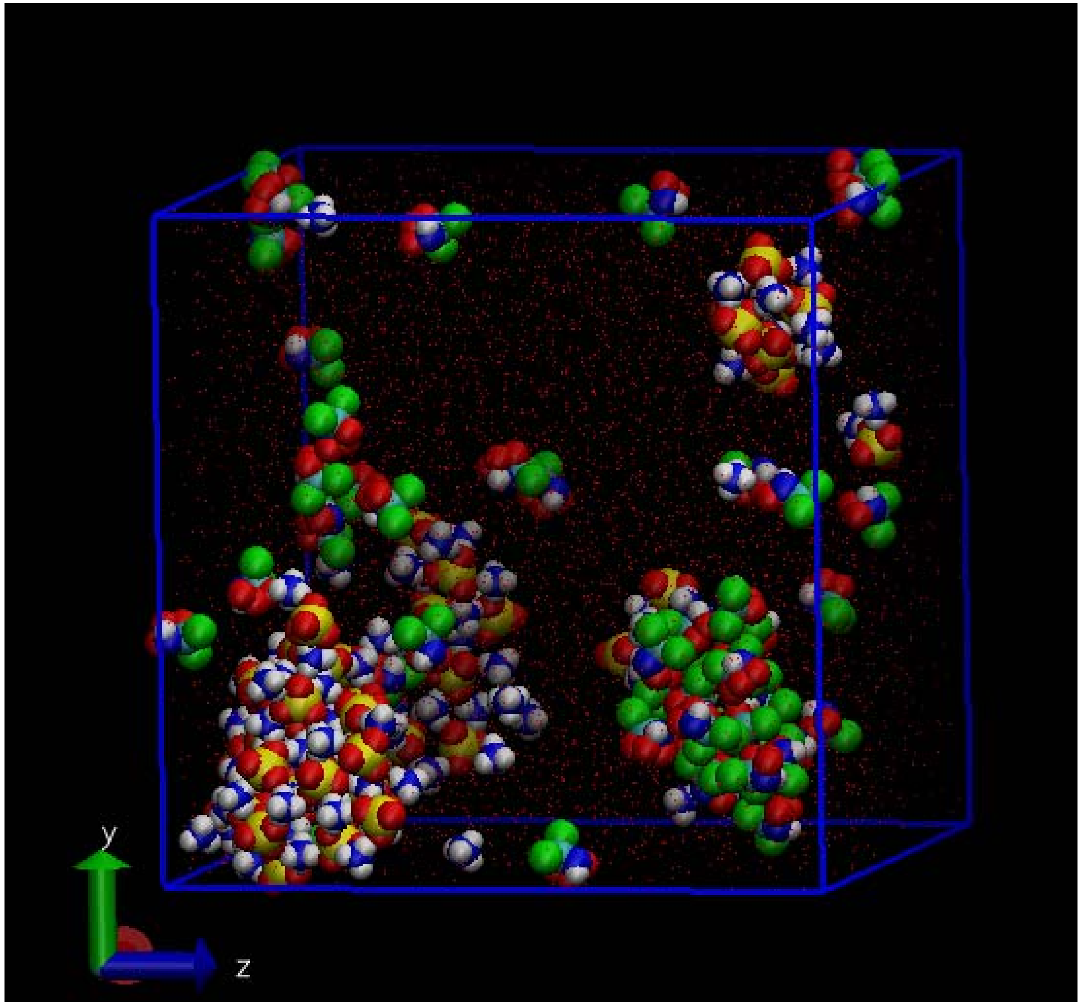

Supplementary Figure S9 The snapshot of distribution of the equilibrium configuration in the P3-$(NH_4)_2SO_4$-H_2O system.

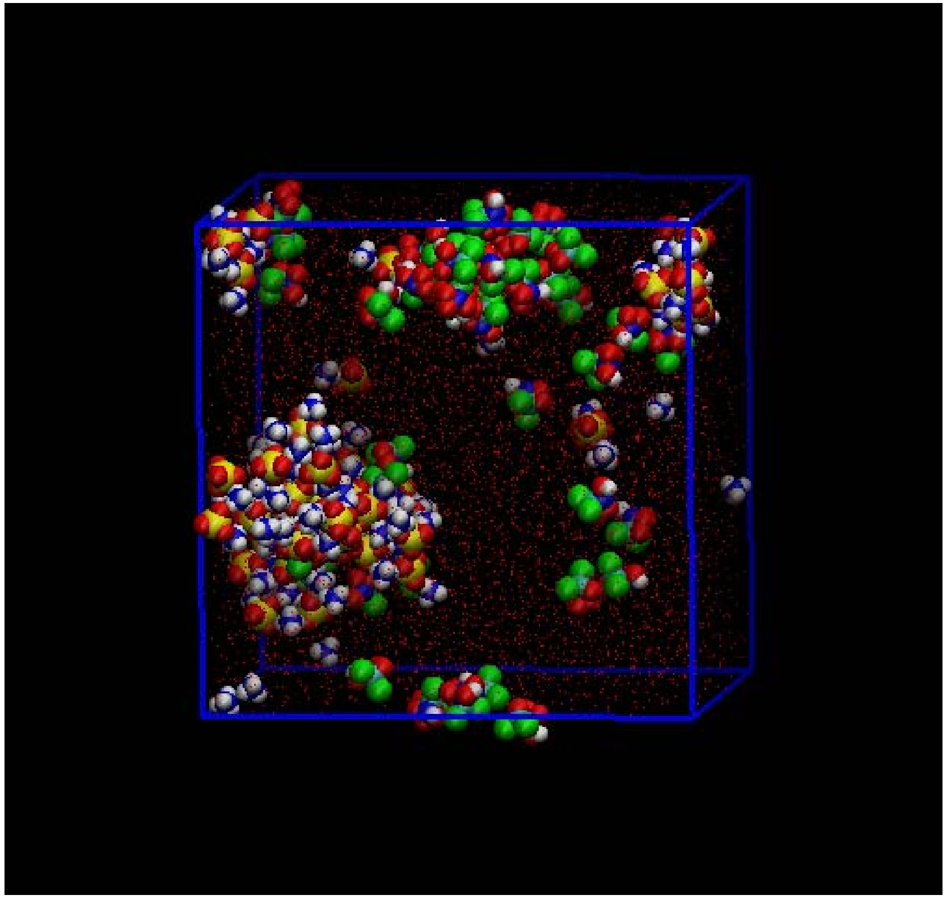

Supplementary Figure S10 The snapshot of distribution of the equilibrium configuration in the P6-$(NH_4)_2SO_4$-H_2O system.

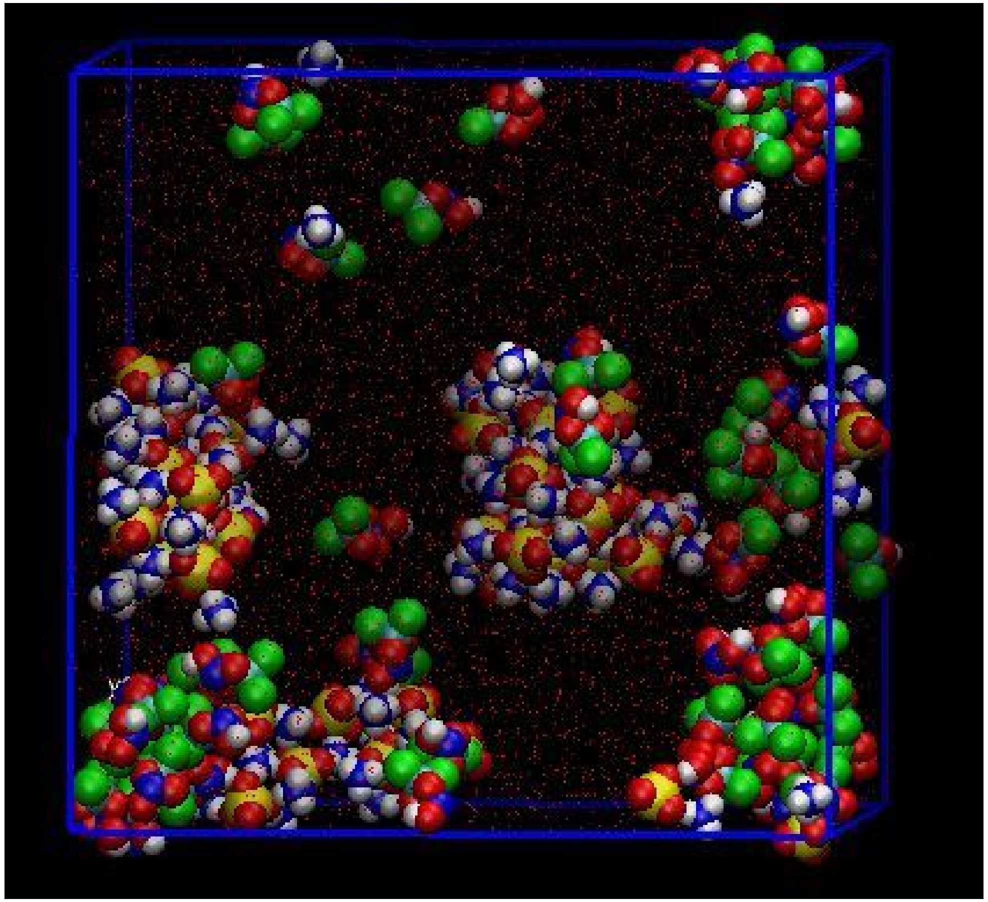

Supplementary Figure S11 The snapshot of distribution of the equilibrium configuration in the P7-$(NH_4)_2SO_4$-H_2O system.

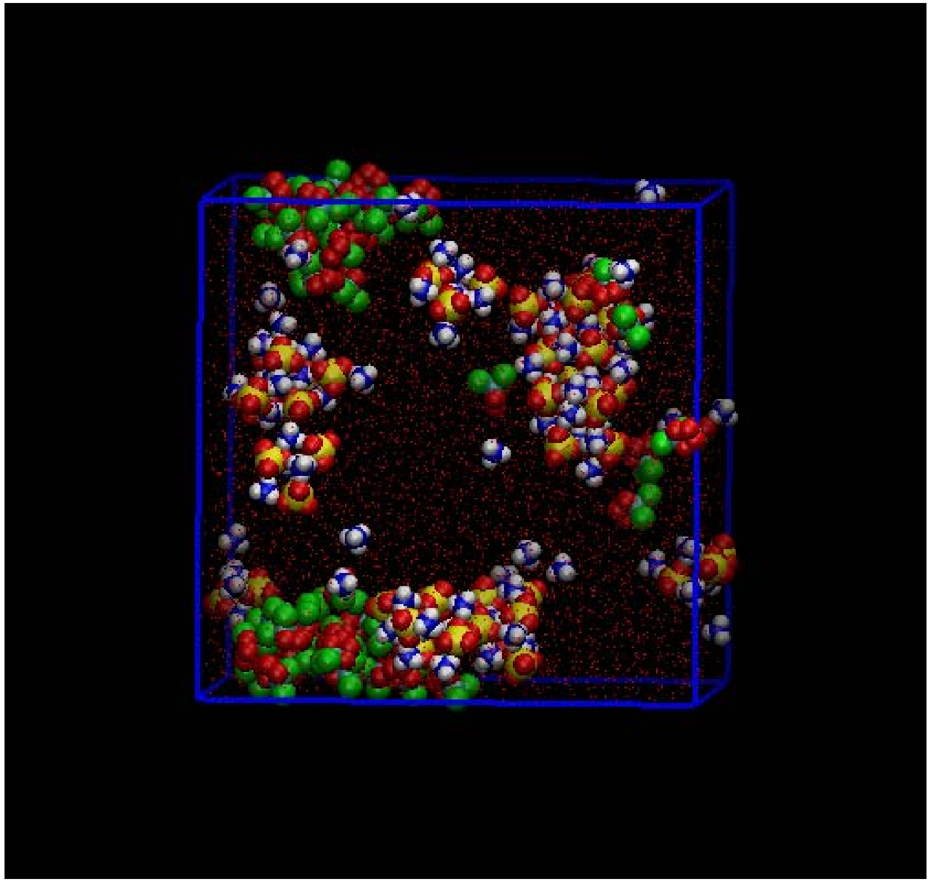

Supplementary Figure S12 The snapshot of distribution of the equilibrium configuration in the P10-$(NH_4)_2SO_4$-H_2O system.

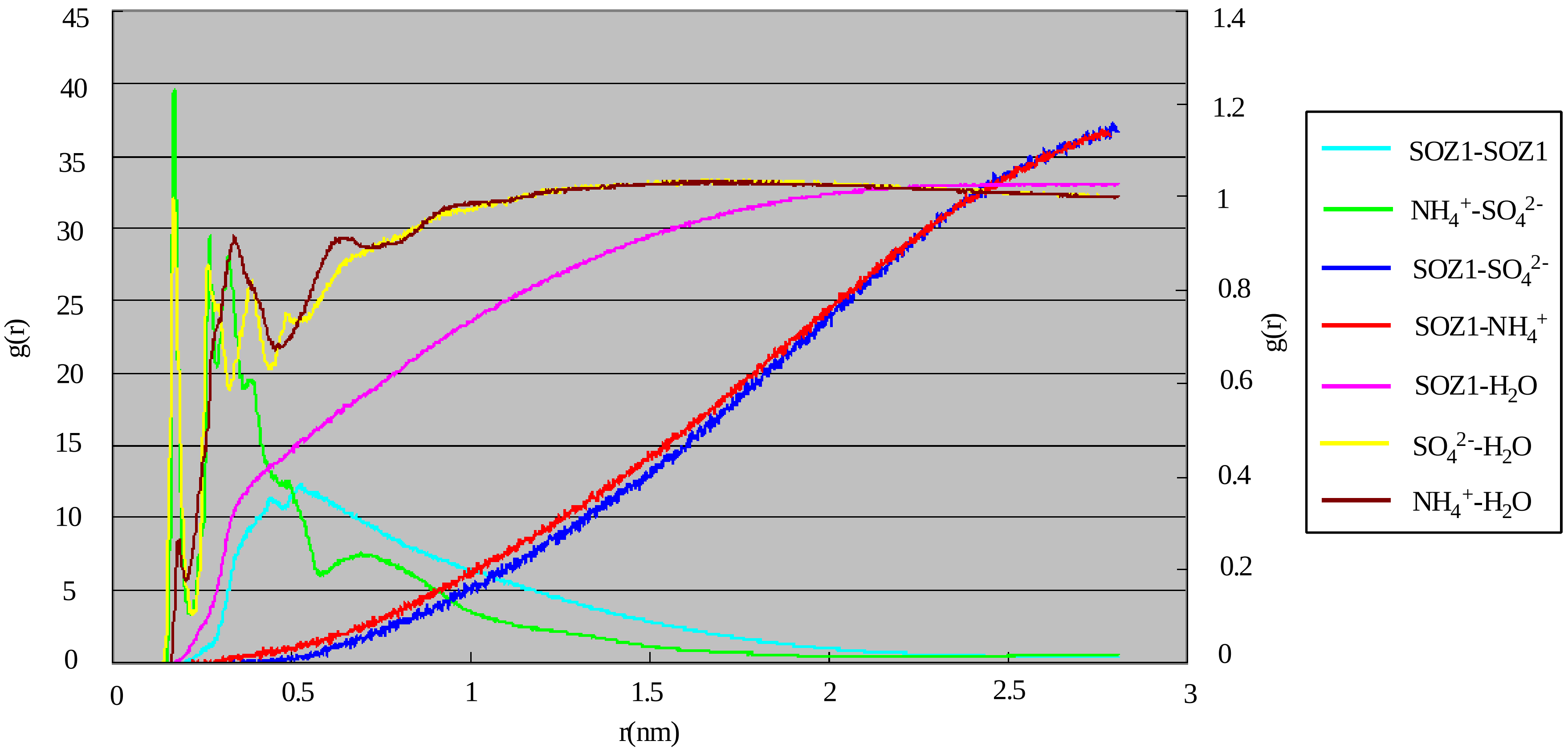

Supplementary Figure S13 The radial distribution functions for the main particles in the SOZ1-$(NH_4)_2SO_4$-H_2O system.

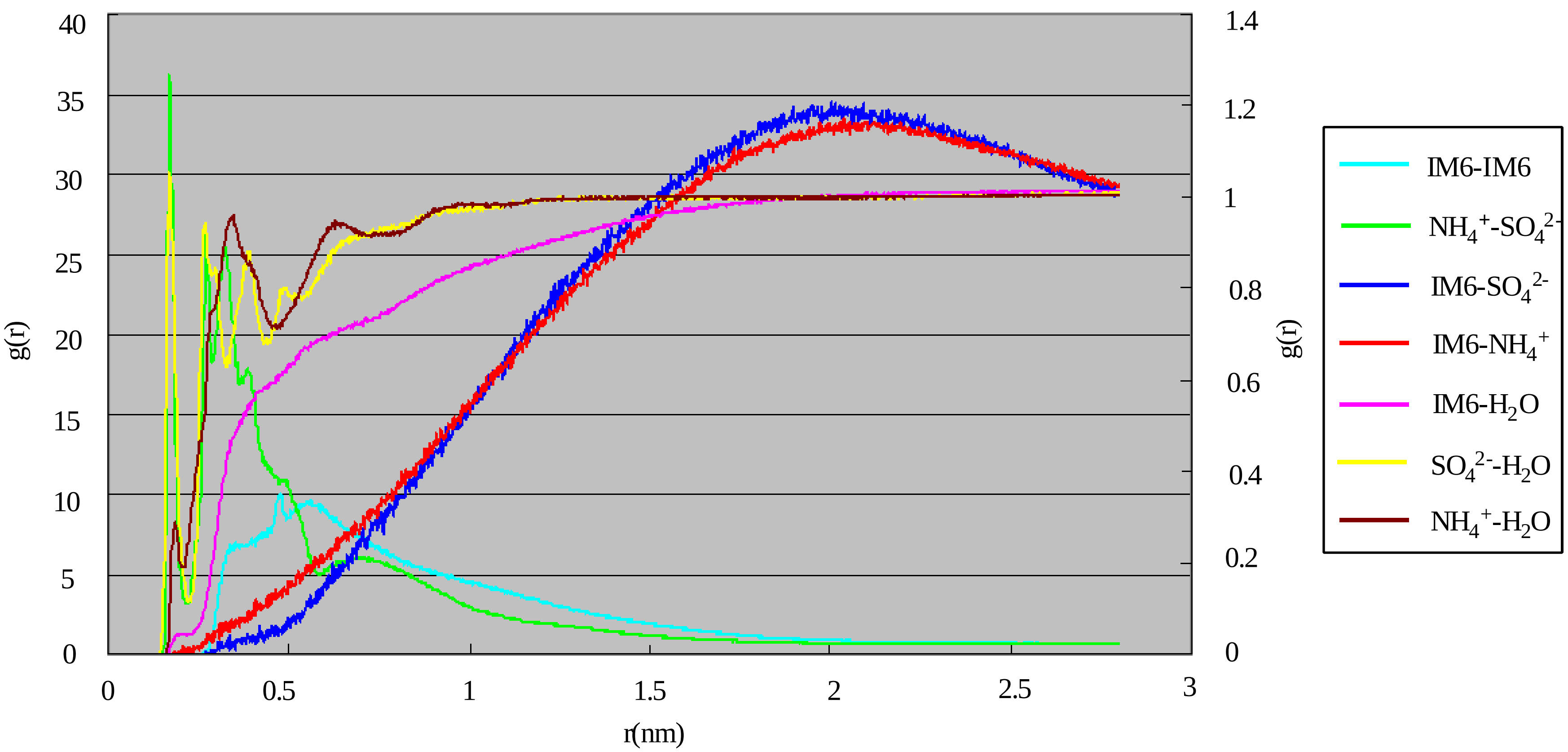

Supplementary Figure S14 The radial distribution functions for the main particles in the IM6-$(NH_4)_2SO_4$-H_2O system.

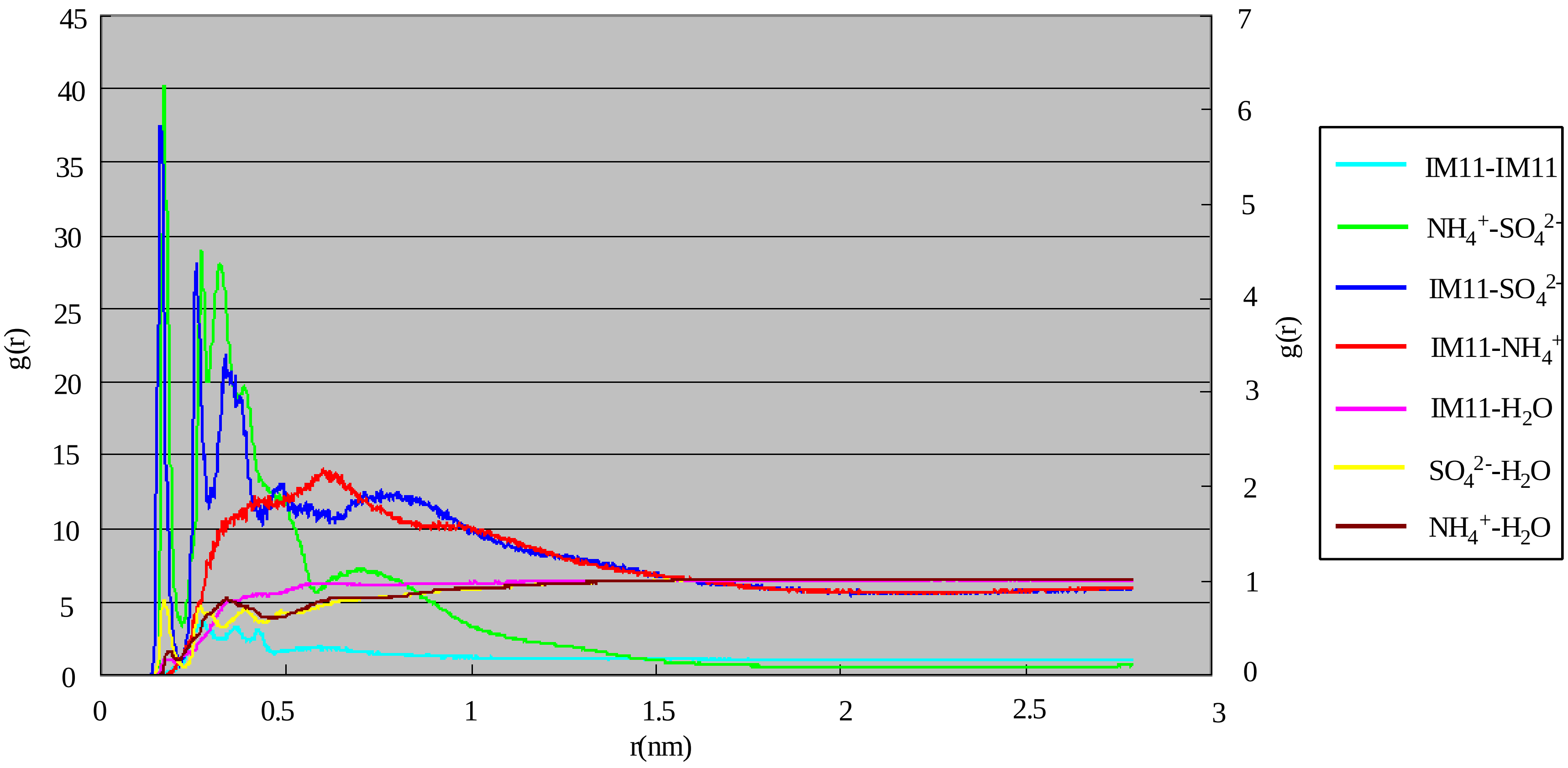

Supplementary Figure S15 The radial distribution functions for the main particles in the IM11-$(NH_4)_2SO_4$-H_2O system.

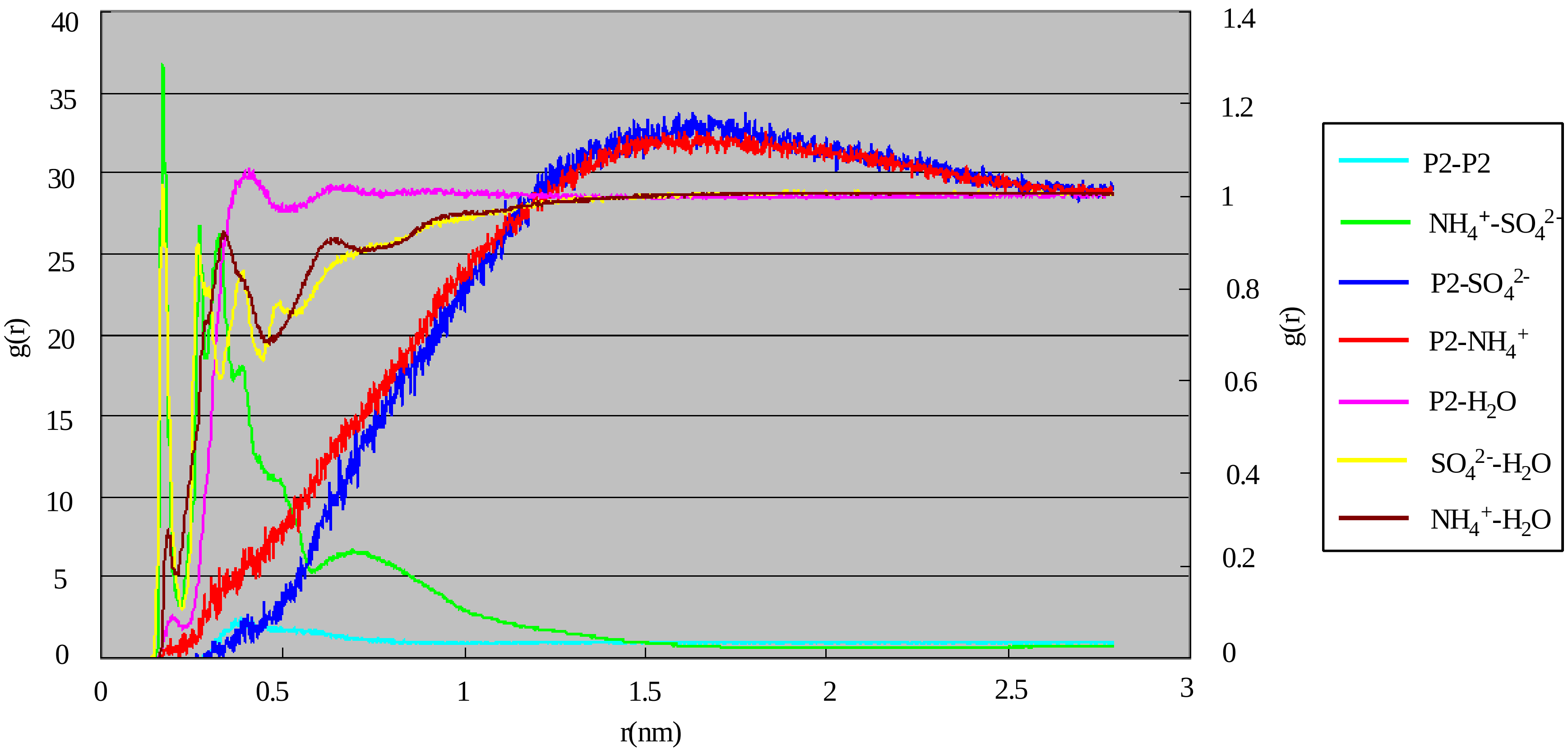

Supplementary Figure S16 The radial distribution functions for the main particles in the P2-$(NH_4)_2SO_4$-H_2O system.

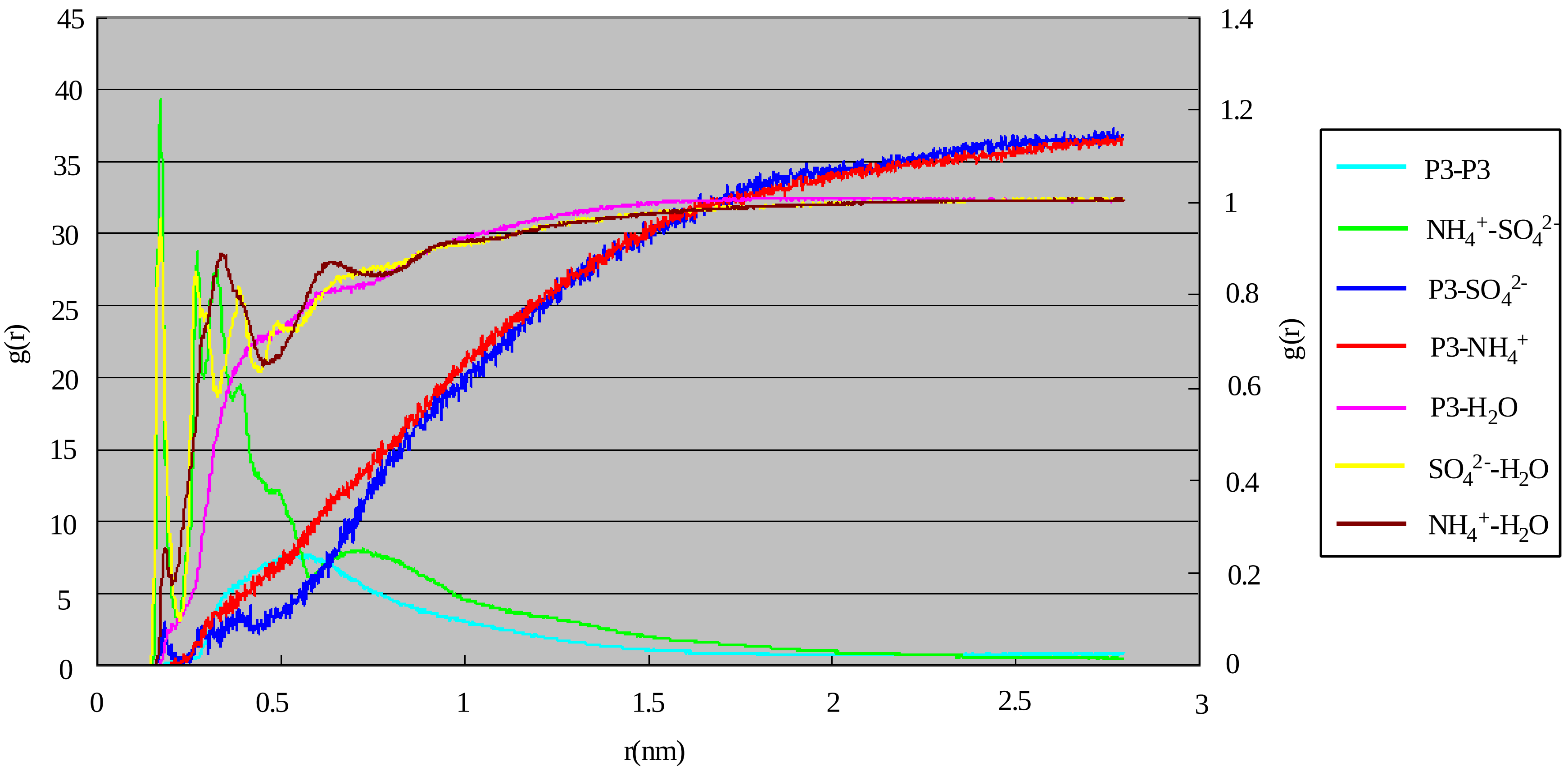

Supplementary Figure S17 The radial distribution functions for the main particles in the P3-$(NH_4)_2SO_4$-H_2O system.

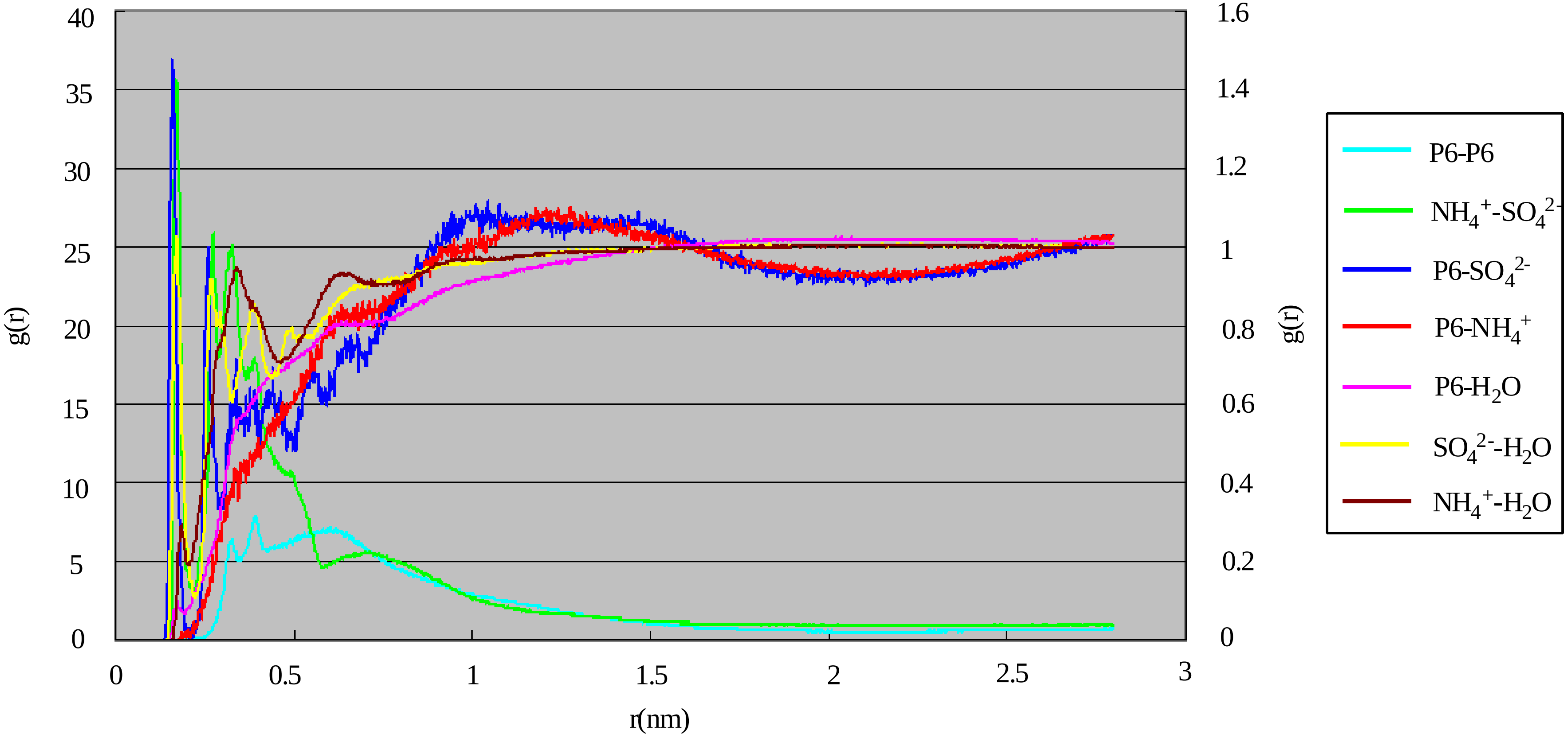

Supplementary Figure S18 The radial distribution functions for the main particles in the P6-$(NH_4)_2SO_4$-H_2O system.

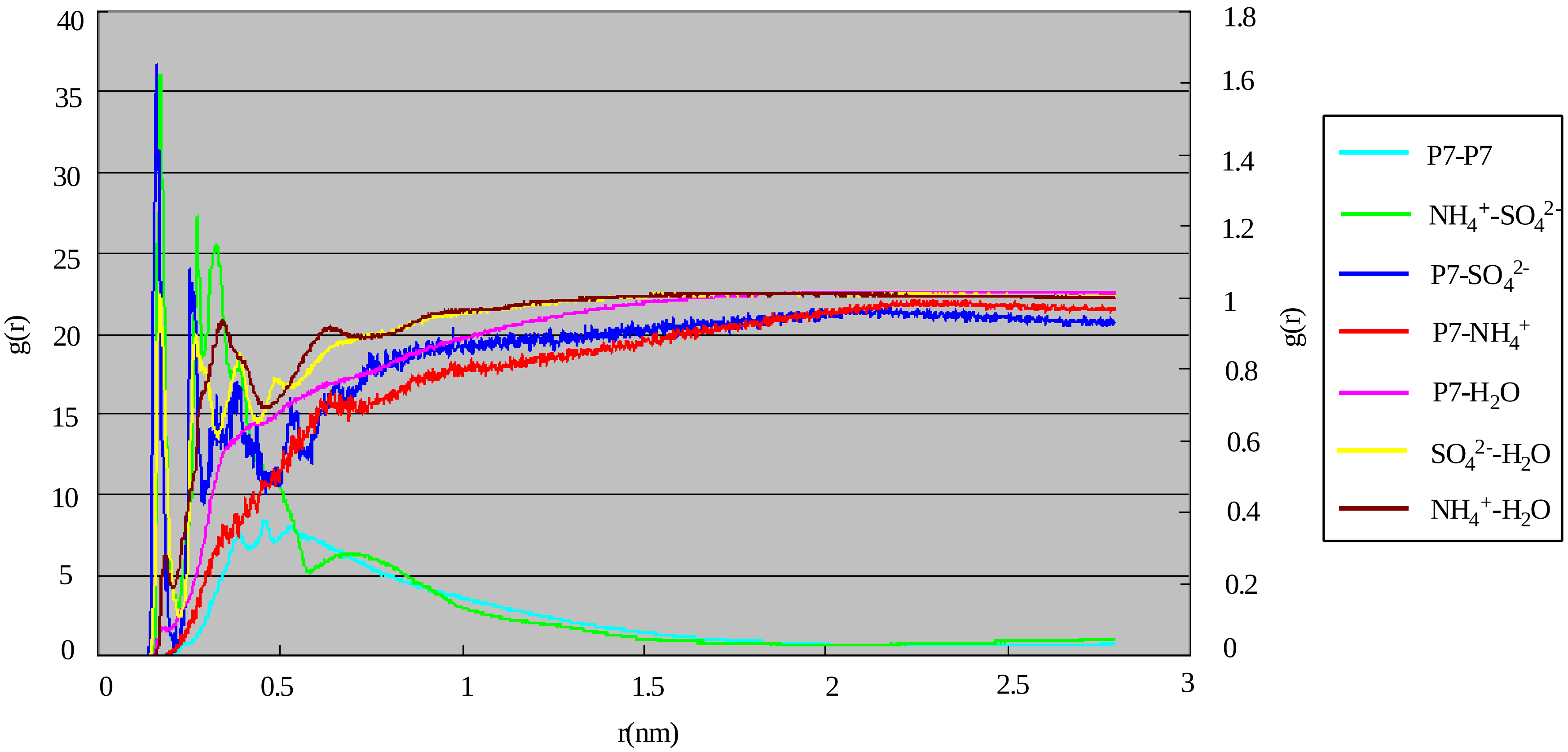

Supplementary Figure S19 The radial distribution functions for the main particles in the P7-$(NH_4)_2SO_4$-H_2O system.

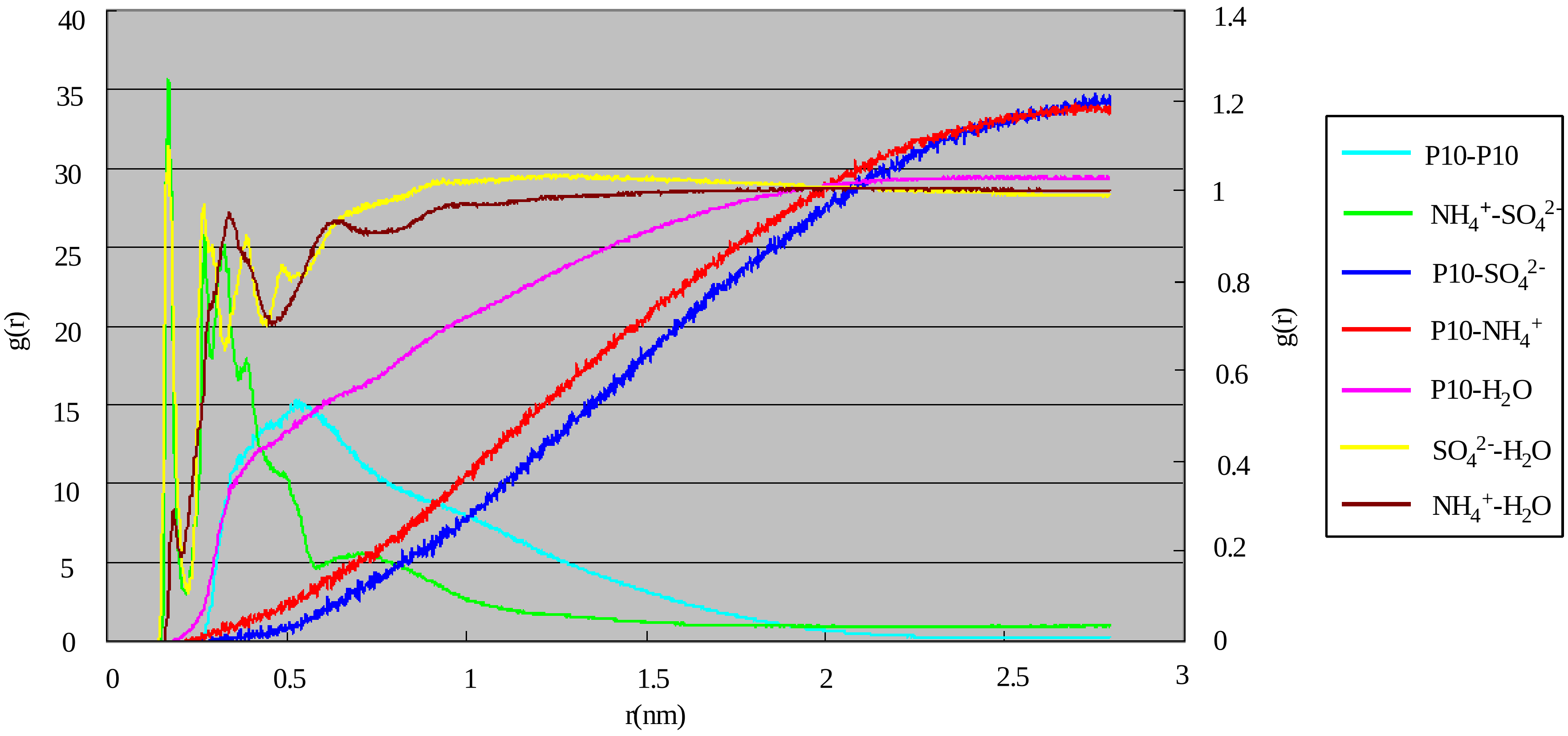

Supplementary Figure S20 The radial distribution functions for the main particles in the P10-$(NH_4)_2SO_4$-H_2O system. (The left axis represents the g(r) of groups POZ-POZ and NH_4^+-SO_4^{2-}, the right axis represents the g(r) of the other groups)
